# Mineração de textos e dados na pesquisa em saúde: reflexões sobre direitos autorais

**DOI:** 10.1590/0102-311XPT169023

**Published:** 2024-05-20

**Authors:** Allan Rocha de Souza, Luca Schirru, Miguel Bastos Alvarenga

**Affiliations:** 1 Programa de Pós-graduação em Políticas Públicas, Estratégias e Desenvolvimento, Universidade Federal do Rio de Janeiro, Rio de Janeiro, Brasil.; 2 Instituto Brasileiro de Direitos Autorais, Rio de Janeiro, Brasil.; 3 Centre for IT & IP Law (KU Leuven), Leuven, Belgium.

O uso de sistemas informáticos para auxiliar na coleta, organização e análises de grandes volumes de dados e textos e, neste contexto, as técnicas de mineração de textos e dados (*text and data mining -* TDM) ^1^ são centrais às pesquisas contemporâneas intensivas em dados, como aquelas conduzidas no combate à pandemia de COVID-19 ^2^^,^^3^^,^^4^^,^^5^^,^^6^^,^^7^^,^^8^. A par de questões éticas referentes à apropriação dos dados de pesquisa ^8^, os direitos autorais impõem desafios específicos ao uso de TDM, tanto em razão dos objetos sobre os quais incide a proteção, como por sua extensão temporal, amplo escopo de direitos atribuídos e escassez de limitações expressas. Em razão de sua importância para a pesquisa, nos concentramos aqui justamente nestes obstáculos e os movimentos de regulação em curso.

Os direitos autorais não protegem fatos, informações ou dados, e nem mesmo o conteúdo de uma obra, pois seu objeto de proteção é a forma literária ou artística em que são expressos, comunicados. No entanto, quando estes elementos são agrupados, organizados ou sistematizados em uma base ou banco de dados que seja minimamente original com relação à seleção, organização ou disposição de seu conteúdo, este material passa a ter seu acesso e utilização controlado pelo titular deste conjunto, então protegido por direitos autorais ^9^^,^^10^^,^^11^.

Ademais, a proteção jurídica por direitos autorais das bases e bancos de dados é apenas uma dentre as camadas de controle sobre o acesso e uso a dados e informações ^11^. Há uma segunda camada, composta por restrições tecnológicas ao acesso ou uso de bases de dados ^12^, e, ao menos, ainda, uma terceira, sob a forma de proibições legais de burla desses mecanismos, mesmo quando não há violação direta de qualquer direito incidente sobre o material ^13^. Isto impacta diretamente atividades essenciais à pesquisa, como verificação, reprodutibilidade e comunicação dos resultados, ao permitir ou limitar quem pode fazer que tipo de pesquisa, utilizando que tipo de material e em que condições ^14^^,^^15^^,^^16^. Isto se sobressai na TDM, que necessita da maior disponibilidade de fontes possível - as quais nem sempre existem em acesso aberto. Assim, restringir-se a esse tipo de fonte nesse tipo de método pode levar ao exacerbamento de vieses nos resultados ^15^.

Há em curso uma demanda por reformas normativas no âmbito global, diante da extensão normativa, tecnológica e contratual dos poderes de controle pelos titulares, que impõe desafios jurídicos a inúmeras atividades de pesquisas intensivas em dados ^17^^,^^18^^,^^19^, o que é especialmente sentido nos países do Sul Global ^19^^,^^20^^,^^21^. Diante disso, diversos sistemas jurídicos iniciaram, na última década, reformas em suas legislações de direitos autorais para incluir normas visando assegurar a legalidade da TDM ^22^, a exemplo da União Europeia ^23^^,^^24^, e de países como Japão ^25^^,^^26^ e Singapura ^27^. 

No Brasil, há o reconhecimento dos potenciais desafios e oportunidades quanto ao uso de tecnologias intensivas em dados nos textos da Estratégia Brasileira de Inteligência Artificial (EBIA) ^28^ e na Estratégia Nacional de Propriedade Intelectual (ENPI) ^29^. Contudo, até agora, a discussão mais aprofundada sobre TDM ocorreu no âmbito dos debates na Comissão de Juristas de Inteligência Artificial (CJUSBIA), responsável por subsidiar a elaboração de substitutivo do *Projeto de Lei* (PL) sobre Inteligência Artificial ^30^.

O relatório final da CJUSBIA, convertido agora no *PL nº 2.338/2023* do Senado Federal, propõe uma definição de mineração de textos e dados para fins normativos, além de uma limitação (ou exceção) aos direitos autorais do titular de bancos e bases de dados, bem como de outras obras envolvidas em tal processo ^31^. A limitação proposta no art. 42 do *PL nº 2.338/2023* é bastante clara e delimitada em seu escopo: apenas autoriza o acesso e uso por entidades cuja missão é conectada ao interesse público - por exemplo, instituições de pesquisa e de jornalismo - e na extensão necessária para os objetivos pretendidos. A limitação também deixa claro que o uso ali referenciado é aquele que, no que tange a obras protegidas, não implique na criação de produtos concorrentes ou que venham, de alguma forma, impactar na exploração normal das obras utilizadas, por exemplo ^31^.

Alguns defendem que sequer seria preciso uma limitação para mineração de textos e dados ^24^^,^^32^, por ser um uso não-expressivo ^33^^,^^34^; ou, porque o que se extrai das obras - padrões e elementos factuais, por exemplo - não integraria o escopo de proteção dos direitos autorais ^24^^,^^32^^,^^34^. Contudo, múltiplos projetos já recorreram ao uso direto de conteúdo protegido por direitos autorais e causaram disputas judiciais, mesmo quando o material havia sido acessado legalmente ^33^. Assim, uma previsão normativa expressa é de extrema importância para garantir segurança jurídica às organizações e instituições de pesquisa, jornalismo, museus, arquivos, bibliotecas e também de seus servidores, na satisfação de suas funções e missões institucionais no contexto contemporâneo da pesquisa, inovação e desenvolvimento tecnológico ^35^. 

Mais amplamente, a discussão sobre a legitimidade e importância da TDM para fins de pesquisa tem sido acompanhada de um debate mais amplo sobre o reconhecimento, contornos e efetividade de um direito à pesquisa enquanto garantia fundamental. Regularmente atrelado nos textos normativos, seja ao direito à educação e de acesso ao conhecimento (art. 27 da *Declaração Universal dos Direitos Humanos* e arts. 206, II e 208, V da *Constituição Federal*) ^36^^,^^37^, ou, mais amplamente, à ciência e inovação (art. 218 e seguintes da *Constituição Federal*) ^37^, suas particularidades demandam independência e autonomia própria, e a elaboração de sua estrutura legal, funções normativas e efeitos jurídicos próprios ainda está em construção ^38^.

No entanto, a concreção e realização deste direito não têm acompanhado sua relevância social. A recente pandemia expôs a centralidade e a importância da pesquisa e da ciência no funcionamento da sociedade. Os anos recentes nos explicitaram a vulnerabilidade política e social a que as atividades de pesquisa estão sujeitas. Reconhecer a legitimidade da mineração de textos e dados - especialmente, mas não só para fins de pesquisa - é um passo importante e necessário, embora não suficiente, na elaboração e consolidação do direito à pesquisa, que entendemos como axial para fomentar a inovação, o desenvolvimento econômico e autonomia tecnológica do país.
